# Why have extensive efforts to improve adolescents’ physical fitness seen limited success? A mediation analysis of physical activity enjoyment, physical activity and physical fitness

**DOI:** 10.3389/fpsyg.2025.1572826

**Published:** 2025-07-31

**Authors:** Jiale Peng, Wenlang Yu, Wenxing Wang, Leyi Wang, Wei Shan, Hong Ren

**Affiliations:** ^1^College of Physical Education, Guangdong University of Education, Guangzhou, China; ^2^School of Sport Science, Beijing Sport University, Beijing, China; ^3^Key Laboratory of Exercise and Physical Fitness (Beijing Sport University), Ministry of Education, Beijing, China; ^4^Sports Coaching College, Beijing Sports University, Beijing, China; ^5^China Institute of Sport and Health Science, Beijing Sport University, Beijing, China

**Keywords:** exercise, exercise enjoyment, teenager, mediation analysis, health promotion

## Abstract

**Objective:**

Investigate the association of physical activity enjoyment (PAE), physical activity (PA) and physical fitness (PF) among adolescents using a mediation model.

**Methods:**

845 students (420 males, 425 females) were selected via stratified random cluster sampling. Demographic data were gathered through a structured questionnaire. Adolescent PAE was evaluated using the Physical Activity Enjoyment Scale, while PA was quantified with the Physical Activity Rating Scale (PARS-3). PF was assessed on-site in accordance with the guidelines outlined in the National Physical Fitness Standards for Students. To analyze the relationships among these variables, correlation analyses and mediation techniques were employed.

**Results:**

The correlation analysis revealed that PAE was positively correlated with PA (*r* = 0.622, *p* < 0.001) and PF (*r* = 0.291, *p* < 0.001), PA was positively correlated with PF (*r* = 0.256, *p* < 0.001). According to the results of the mediation analysis, PAE significantly predicts PA (standardized *β* coefficient = 0.5274, *p* < 0.001) and PF (standardized *β* coefficient = 0.2660, *p* < 0.001) in a positive direction. PA significantly predicts PF (standardized *β* coefficient = 0.1878, *p* < 0.001). Bootstrap-generated confidence intervals (CI) revealed a significant indirect effect for PAE on PF (*β* = 0.269, 95% CI = 0.147 to 0.389, *p* < 0.05) and a significant direct effect for PAE on PF (*β* = 0.721, 95% CI = 0.513 to 0.930, *p* < 0.001). According to the proportion of effect calculation, the mediation effect was 27.2%.

**Conclusion:**

PAE is positively associated with PF, with PA partially mediated the relation between PAE and PF. This suggests that neglecting the development of PAE in adolescents may undermine efforts to improve PF through increases in PA levels.

## Introduction

Adolescents’ physical fitness (PF) plays a crucial role in their personal development and social integration. PF in adolescence has a profound impact on long-term health in adulthood (Meyer et al., 2021; Fraser et al., 2017; Leong et al., 2015; Bezold et al., 2014). Extensive efforts have been undertaken to enhance the PF of adolescents. Current intervention studies predominantly employ strategies such as exercise interventions, health education, health promotion, and management strategies. These interventions are typically delivered across diverse settings, including schools, households, and extracurricular sports organizations (Song et al., 2012; Liu et al., 2019; Bai et al., 2018; Chen et al., 2019; Neil-Sztramko et al., 2021; Schmidt et al., 2020; Zahner et al., 2006; Kolle et al., 2020; Kriemler et al., 2011; Ørntoft et al., 2016). Current public health guidelines for children and adolescents advocate for a minimum of 60 min of daily moderate-to-vigorous physical activity (MVPA), supplemented by muscle and bone strengthening activities at least 3 days per week (Chaput et al., 2020). In fact, research has shown that most adolescents fail to meet current physical activity (PA) recommendations (Guthold et al., 2020; Kalman et al., 2015), and that the PF of Chinese adolescents is on a clear downward trend. Insufficient PA and declining physical health among adolescents continue to pose a growing public health concern.

Studies have indicated that a lack of enjoyment in exercise is associated with insufficient physical activity (Bai et al., 2018; Chen et al., 2019; Zhang et al., 2021) and lower levels of physical fitness (Vanden Bosch et al., 2014; Prochaska et al., 2003; Woods et al., 2012; Jin et al., 2018), highlighting the critical role of enjoyment in exercise outcomes. Physical activity enjoyment (PAE) is essential for stimulating intrinsic motivation for physical activity (PA), which is driven by the activity itself rather than external pressures or rewards (Gao, 2023; Remmers et al., 2015). According to Self-Determination Theory (SDT) (Deci and Ryan, 2000), human behavior is motivated by both intrinsic and extrinsic factors, but highly autonomous intrinsic motivation is more effective in regulating behavior and achieving positive outcomes (Teixeira et al., 2012). Therefore, enhancing PAE may not only boost intrinsic motivation but also contribute to long-term engagement in physical activity (Remmers et al., 2015), ultimately improving physical fitness (PF).

The suboptimal PF development observed among adolescents may stem from an overemphasis on external factors in the design of interventions and educational strategies, coupled with a lack of focus on enhancing intrinsic motivation. By prioritizing intrinsic motivation through enjoyment, interventions can promote sustained physical activity behavior, thereby addressing the challenges of inadequate PF development.

This study seeks to examine the mechanisms by which PA and PAE impact adolescents’ PF, employing a mediated effects model. Accordingly, the following two research hypotheses are proposed: PAE positively predicts adolescents’ PF (H1); PAE influences adolescents’ PF through the mediating role of PA (H2).

## Objects and methods

### Study design

This cross-sectional survey was conducted in Shanxi Province, China, between October and November 2023. Stratified random sampling was employed to select the study population. Schools were chosen based on the level of socio-economic development, with one junior high school (grades 7 to 9) and one senior high school (grades 10 to 12) selected from areas of higher and lower economic development, respectively. Within each school, two to three classes were randomly selected from each grade level using a cluster sampling approach.

**Figure 1:**
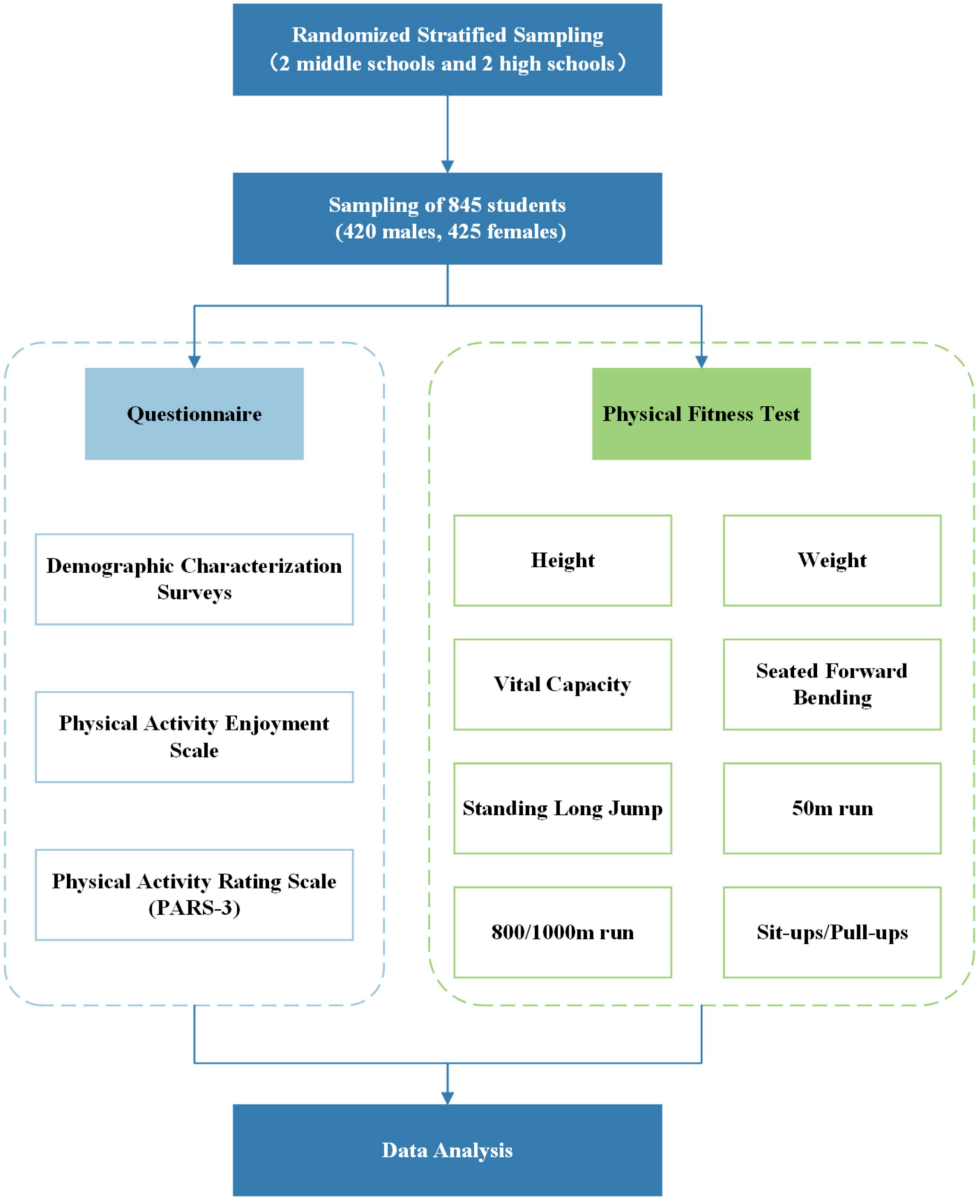
Flow chart of the research.

All participants completed the PF test and self-reported questionnaires. Informed consent was obtained from the schools, the students, and their parents prior to the survey. The study design is shown in Figure 1. This study was approved by the Sport Science Experiment Ethics Committee of Beijing Sport University (No. 2020128H).

### Participants

A sample of 845 students (420 males, 425 females) were ultimately included in this study. Among them, 428 (50.7%) were junior high school students and 417 (49.3%) were senior high school students, with an average age of 14.8 ± 1.7 years (ranging from 12 to 19 years). Sample size estimation was based on prior simulation studies indicating that detecting small-to-medium mediation effects requires at least 500 participants with adequate power (Fritz and MacKinnon, 2007). The final sample (*N* = 845) met this criterion.

### Method

#### Demographic characteristics

Basic information about the survey respondents, including age, gender, and grade, was collected through an offline questionnaire.

#### Physical activity enjoyment scale

Adolescent PAE in this study was investigated using the Physical Activity Enjoyment Scale (Ye et al., 2010), a Chinese version of the scale adapted from Kendzierski and DeCarlo’s (1991) Physical Activity Enjoyment Scale (PACES). The scale consists of five items rated on a 5-point Likert scale, with a score of 1 indicating complete noncompliance with the statement and a score of 5 indicating complete compliance. The scores for each item were summed to obtain a total scale score, with higher scores indicating higher levels of PAE. The internal consistency coefficient of the scale for the data of this survey was 0.93.

#### Physical activity rating scale (PARS-3)

PA was measured using the PA Rating Scale (PARS-3) (Liang, 1994). The scale assesses the amount of PA in terms of intensity, duration, and frequency of participation. Intensity is rated on a 5-point scale based on subjective perception, ranging from 1 (“light exercise”) to 5 (“intense and prolonged exercise with shortness of breath and profuse sweating”). Frequency is rated from 1 (“less than once a month”) to 5 (“once a day”). Duration is rated from 0 (“less than 10 min”) to 4 (“more than 60 min”). The total PA score is calculated by multiplying the scores for intensity, duration, and frequency, yielding a range from 0 to 100. PA levels are classified as follows: a score of ≤19 indicates low activity; 20–42 indicates moderate activity; and ≥43 indicates high activity.

#### Physical fitness test

The measurements included Body Mass Index (BMI), Vital Capacity (measured using FP-FH808 spirometer, Fairplay, China), sit-and-reach test, standing long jump, 50 m run, 800 m run (for females) or 1,000 m run (for males), and sit-ups (for females) or pull-ups (for males). All tests were conducted using identical instrument models to ensure measurement consistency across participants. According to the National Student Physical Health Standards of Students, each indicator was measured and scored based on the standard’s criteria, with the total PF score calculated by summing the weighted scores of all indicators (Ministry of Education of the People's Republic of China, n.d.).

### Quality control

Both questionnaires and field tests were included in the study. Before completing the questionnaires, students received standardized training and instructions from their teachers. Field tests were conducted by trained research team members, assisted by a score recorder. Scores were cross-verified by team members and the recorder to ensure accuracy. Each student’s score was entered into the system twice to prevent errors. In cases where discrepancies arose between entries, the system flagged an error, prompting a re-entry of the score.

### Statistical methods

Statistical analysis was performed using SPSS 27.0 software. Non-normally distributed continuous variables were described using median (interquartile range, IQR). Gender and grade differences in PAE, PF and PA were assessed using the Mann–Whitney U test, Kruskal-Walli’s test, and chi-square test as appropriate. Spearman’s correlation analysis was used to explore the relationships among the variables of PAE, PF and physical activity. The mediating role was tested using Model 4 of PROCESS, with 5,000 Bootstrap samples to obtain standard errors of parameter estimates and Bootstrap confidence intervals, which were considered significant if they did not include zero. *p* < 0.05 was considered statistically significant.

## Results

### Basic characteristics of adolescents’ PAE, PA and PF

The basic characteristics of the participants and the distribution of PAE, PA and PF are shown in Table 1. Statistically significant differences were observed in PAE (*p* < 0.001), PF (*p* = 0.002) and PA (*p* < 0.001) among adolescents of different genders. Overall, boys had higher levels of PA and PAE compared to girls, while girls scored higher in PF than boys. Across different grades, significant differences were found in PF (*p* < 0.001) and PA (*p* = 0.041). Across different grades, significant differences were found in PF (*p* < 0.001) and PA (*p* = 0.041). PF showed a general increase from the first year, peaking in the third year, then significantly declining in the first year of high school, and remaining lower in the subsequent years. No significant differences were found in PAE across different grades.

**Table 1:** Basic characteristics of the participants and distribution of PAE, PA and PF.

*N*	PAE	PF	PA [*N* (%)]		
			Low	Moderate	High
General (845)	17.0 (14.0, 21.0)	71.1 (62.4, 78.5)	501 (59.3)	212 (25.1)	132 (15.6)
Genders					
Male (420)	19.0 (15.0, 23.0)	69.9 (61.4,76.7)	188 (44.8)	120 (28.6)	112 (26.7)
Female (425)	15.0 (13.0, 18.0)	73.2 (64.2,79.8)	313 (73.6)	92 (21.6)	20 (4.7)
*Z*/*χ^2^*	−10.28	−3.16	98.98		
*p*	<0.001^**^	0.002^**^	<0.001^**^		
Grade					
Grade 7 (146)	16.0 (14.0, 20.3)	69.2 (58.9, 76.2)	105 (71.9)	23 (15.8)	18 (12.3)
Grade 8 (140)	17.0 (14.0, 21.0)	73.3 (66.7, 80.2)	80 (57.1)	37 (26.4)	23 (16.4)
Grade 9 (142)	16.5 (14.0, 20.0)	79.5 (70.4, 84.5)	72 (50.7)	43 (30.3)	27 (19.0)
Grade 10 (128)	16.0 (14.0, 21.0)	69.2 (61.0, 76.2)	79 (61.7)	32 (25.0)	17 (13.3)
Grade 11 (137)	17.0 (15.0, 2.0)	70.0 (62.6, 77.1)	71 (51.8)	40 (29.2)	26 (19.0)
Grade 12 (152)	18.0 (15.0, 20.0)	67.4 (55.7, 74.2)	94 (61.8)	37 (24.3)	21 (13.8)
*H*/ *χ^2^*	4.601	93.72	18.91		
*P*	0.466	<0.001^**^	0.041^*^		

*p < 0.05, **p < 0.001.

### Relationship between PAE and PA and PF

Spearman’s correlation analysis revealed that PAE was positively correlated with PA (*r* = 0.622, *p* < 0.001) and PF (*r* = 0.291, *p* < 0.001), PA was positively correlated with PF (*r* = 0.256, *p* < 0.001) (see Table 2).

**Table 2:** Correlation between PAE, PA and PF.

Variable	PAE	PA	PF
PAE	1		
PA	0.622^**^	1	
PF	0.291^**^	0.256^**^	1

**p < 0.001.

The results of the correlation analysis between PAE and PF indicators for boys and girls are presented in Table 3. Among boys, PAE showed significant positive correlations with BMI (*p* < 0.01), the 50-m run (*p* < 0.01), the 1,000 m/800 m run (*p* < 0.01), the Sit-and-Reach Test (*p* < 0.05), the standing long jump (*p* < 0.01), and pull-ups/sit-ups (*p* < 0.01). Similarly, for girls, PAE demonstrated significant positive correlations with the 50-m run (*p* < 0.01), the 1,000 m/800 m run (*p* < 0.01), the Sit-and-Reach Test (*p* < 0.01), the standing long jump (*p* < 0.01), and pull-ups/sit-ups (*p* < 0.01).

**Table 3:** Correlation between PAE and PF.

Subscale	BMI	Vital capacity	50 m	1,000 m/800 m	Sit and Reach Test	Standing long jump	Pull-ups/sit-ups	Total score of PF
PAE (male)	0.193^**^	0.015	0.435^**^	0.332^**^	0.099^*^	0.427^**^	0.282^**^	0.406^**^
PAE (female)	0.084	−0.016	0.300^**^	0.259^**^	0.220^**^	0.327^**^	0.129^**^	0.302^**^

*p < 0.05, **p < 0.001.

### Mediation effect analysis

Based on the preceding analysis, PAE, PA and PF are significantly correlated in pairwise relationships, fulfilling the prerequisites for testing mediation effects. Controlling for gender and grade, a mediation model was constructed with PAE as the independent variable, PA as the mediator, and PF as the dependent variable. According to the results of the mediation analysis, PAE significantly predicts PA (standardized *β* coefficient = 0.5274, *p* < 0.001) and PF (standardized *β* coefficient = 0.2660, *p* < 0.001) in a positive direction. PA significantly predicts PF (standardized *β* coefficient = 0.1878, *p* < 0.001) (Table 4).

**Table 4:** Process test results of mediating effect by stepwise regression method.

Steps	Dependent variable	Independent variable	*R*	*R-sq*	*F*	β^*^	*t*
Step One	PA	PAE	0.6057	0.3668	162.421^***^	0.5274	18.094^***^
Step Two	PF	PAE	0.3954	0.1563	51.940^***^	0.3651	10.851^***^
Step Three	PF	PAE	0.4227	0.1787	45.677^***^	0.2660	6.795^***^
		PA				0.1878	4.773^***^

β^*^ represents the standardized beta coefficient. ***represents statistical significance at *p* < 0.001.

**Figure 2:**
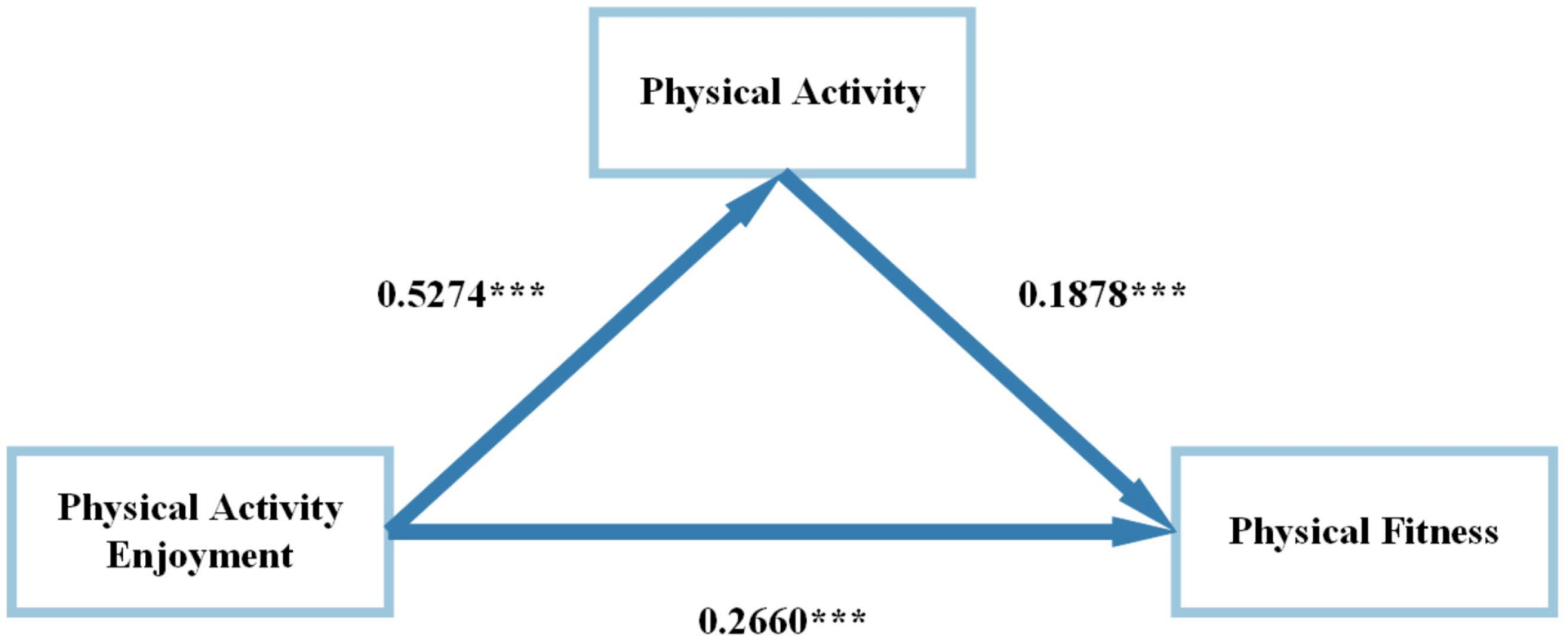
Mediating model diagram. ^***^Represents *p* < 0.001.

In terms of mediation mechanisms (Hypothesis 2), results showed that PA partially mediated the association between PAE and PF. In particular, bootstrap-generated confidence intervals (CI) revealed a significant indirect effect for PAE on PF (*β* = 0.269, 95% *CI* = 0.147 to 0.389, *p* < 0.05) and a significant direct effect for PAE on PF (*β* = 0.721, 95% *CI* = 0.513 to 0.930, *p* < 0.001). The examination of the path coefficients demonstrated that PAE partially mediated the model (Figure 2). According to the proportion of effect calculation, PAE accounted for 27.2% of the total effect (Table 5).

**Table 5:** Bootstrap mediation effect results.

Effect	Effect size	LLCI	ULCI	Effect ratio
Total effect	0.990	0.811	1.169	
Direct effect	0.721	0.513	0.930	72.8%
Indirect effect	0.269	0.147	0.389	27.2%

## Discussion

The present study investigated the relationships among PAE, PA, and PF. The findings demonstrated that PAE is positively associated with PF, with PA partially mediated the relationship between PAE and PF. Traditional PF intervention strategies often focus on increasing PA through coercive measures or external pressures, which subsequently enhance PF. However, the results of this study highlight the importance of prioritizing PAE in intervention strategies to promote PF development in adolescents. By fostering PAE, adolescents can be motivated to actively engage in physical activities and develop an intrinsic enjoyment of sports, leading to long-term, sustainable improvements in PF.

PF stands as one of the most critical indicators of overall health, playing a fundamental role in determining one’s ability to perform daily physical activities and engage effectively in exercise (Ortega et al., 2008). With multiple components, PF includes cardiorespiratory fitness, musculoskeletal strength, endurance, flexibility, agility, balance. PF in adolescents is increasingly recognized as a key determinant of overall health, with strong associations to cardiometabolic risk factors, cognitive performance, bone health, academic achievement, and mental well-being (Blair et al., 1989; Hurtig-Wennlöf et al., 2007; Ortega et al., 2007). Moreover, PF during childhood is closely linked to long-term health outcomes in adulthood, highlighting its critical role in shaping lifelong health trajectories (Ruiz et al., 2009).

Despite growing awareness of its importance, large-scale studies reveal a concerning decline in physical fitness (PF) among adolescents, with an overall downward trend that worsens with age. A comprehensive analysis of 98 studies across 30 European countries, involving 2,779,165 children and adolescents, assessed PF using nine Eurofit tests, which measured balance, muscular strength, muscular endurance, muscular power, flexibility, speed, speed-agility, and cardiorespiratory fitness (CRF). The findings showed that only 78% of boys and 83% of girls met the standards for healthy CRF, with the proportion decreasing markedly as age increased (Tomkinson et al., 2018). Similarly, in six consecutive national surveys conducted between 1985 and 2014, encompassing 1,494,485 Chinese students, PF indicators—including vital capacity, standing long jump, Sit-and-Reach Test, muscular strength, 50-m run, and 1,000 m/800 m run—showed no significant improvement over the three decades. Instead, the results pointed to a steady and troubling decline in Chinese adolescents’ overall PF levels (Dong et al., 2019).

To improve PF among children and adolescents, most current policies emphasize external intervention approaches, such as structured exercise programs and PA promotion initiatives. While some studies have reported that increased PA leads to significant improvements in body composition, CRF, muscular strength, and other PF indicators (Ness et al., 2007; Ekelund et al., 2012). However, several studies have highlighted the limited effectiveness of such external intervention methods in improving PA and PF among children and adolescents (Metcalf et al., 2012; Nooijen et al., 2017). In fact, these studies argue that PA interventions have largely fallen short of achieving significant improvements in key measures such as body mass index (BMI) or body composition (Metcalf et al., 2012).

The current downward trend in adolescent PF further supports this perspective. A plausible explanation for the paradox of intensive interventions failing to improve adolescent PF is that increases in PA driven by external interventions may be offset by reductions in PA elsewhere in adolescents’ daily lives. This observation implies that intervention-specific exercise sessions may merely replace other periods of activity of similar intensity. For instance, participation in after-school activity clubs might displace time that children would otherwise spend playing outdoors or engaging in spontaneous physical activities during the day or week. A study conducted by Metcalf et al. (2012) found that external exercise interventions and PA promotion for adolescents resulted in an increase of only approximately 4 min in their daily walking or running time. Similarly, Nooijen et al. (2017) performed a meta-analysis assessing the effectiveness of PA interventions for obese children and concluded that current strategies were largely ineffective in enhancing PA. These findings highlight the reality that existing PA interventions do not adequately elevate children’s activity levels to make a meaningful impact on PF.

Furthermore, it has been hypothesized that the challenges associated with PA interventions may be linked to their low acceptance among adolescents (Metcalf et al., 2012). Currently, mandatory PA interventions, such as physical education classes and after-school sports programs, are frequently provided to adolescents in schools. However, these activities often do not resonate with students’ preferences. Although the total amount of PA may comply with the standards established by current guidelines and relevant research, the actual outcomes are often unsatisfactory. To effectively enhance adolescent PF, it has been proposed that physical activity-related interventions should focus on reducing inactivity and sedentary behaviors (Nooijen et al., 2017). This approach may be more effective than traditional mandatory exercise programs. A previously underestimated approach to promoting PA is enhancing children’s and adolescents’ access to enjoyable exercise experiences that foster positive emotions (Greule et al., 2024). Engaging in physical activities that are fun and pleasurable is essential, as it can significantly increase participation in a diverse range of physical pursuits. This emphasis on enjoyment not only helps to minimize negative exercise experiences but also combats the development of avoidant behaviors.

In the field of treating overweight and obesity among children and adolescents, a novel intervention perspective has emerged: PAE (Greule et al., 2024). PAE is a positive emotional experience of happiness, pleasure, and joy (Gao, 2023; Chen et al., 2021; Bajamal et al., 2024). The results of this study further validate the positive predictive effect of PAE on PF. The findings indicate that among boys, adolescents with a normal body mass index (BMI) experience significantly more enjoyment in exercise compared to those who are obese. In terms of PF, both boys and girls showed significant positive correlations between PAE and various fitness components, including cardiovascular endurance (800/1000-m run), speed quality (50-m run), lower limb explosive power (standing long jump), flexibility (sit and reach), and muscular endurance (pull-ups/sit-ups). Prochaska et al. (2003) and Gao (2008) found significant positive correlations between physical education enjoyment and cardiovascular endurance among elementary and middle school students. Woods et al. (2012) combined waist circumference, blood pressure, BMI, and cardiovascular endurance data to create an overall health index, revealing a significant positive correlation between PAE and overall health in middle school students. Similarly, Jin et al. (2018) found consistent results in subjective health perception evaluations of children and adolescents with disabilities. Building on numerous past studies, this paper further clarifies the relationship between various PF attributes and PAE, indicating that better speed quality, lower limb explosive power, flexibility, and muscular endurance are associated with higher PAE.

Rooted in Self-Determination Theory (SDT), intrinsic motivation is vital for sustaining certain behaviors (Deci and Ryan, 2000). Individuals engage in exercise primarily because they derive pleasure from it. Moreover, a high degree of self-determined regulation can promote positive affective responses, such as Physical Activity Enjoyment (PAE). As a manifestation of intrinsic motivation, PAE can help individuals maintain an active lifestyle over time. Studies focusing on overweight or obese populations have demonstrated that PAE can enhance physical activity levels by reducing negative exercise experiences and countering the development of avoidance behaviors. The broaden-and-build theory of positive emotions posits that the core function of positive emotions is to enhance an individual’s present actions (Fredrickson, 1998). Therefore, the positive emotions experienced during exercise can effectively promote participation in physical activities. Moreover, humans have an instinct to “maximize pleasure” and “minimize displeasure.” Positive emotions experienced through certain behaviors encourage individuals to repeat those behaviors, whereas negative emotions reduce the likelihood of those behaviors (Gao, 2023). Hence, higher PAE enhances individual participation in exercise and fosters a stronger commitment to continue, thereby accumulating health benefits and ultimately improving PF. This process also ensures a continuous stream of positive emotional experiences, creating a positive feedback loop.

The results from the mediation model analysis in this study suggest that PA partially mediates the relationship between PAE and PF. This indicates that PAE not only directly impacts PF but also indirectly affects it through physical activity. Enjoyment is a key emotional response to PA. It can stem from the experience of exercising, but it also plays a crucial role in determining an individual’s willingness to participate in physical activities. In a study by Klompstra et al. (2022) involving patients with heart failure, it was found that a lack of enjoyment in PA served as a significant barrier to exercise participation. Moreover, PAE was identified as a mediator in the relationship between exercise motivation and actual engagement in PA. It is also noteworthy that a relatively high proportion (59.3%) of participants in our study fell into the low physical activity group. While this may initially appear concerning, it aligns with findings from previous studies. For instance, a global analysis across 146 countries found that 81% of adolescents aged 11–17 did not meet recommended PA levels (Guthold et al., 2020). In China, a study covering universities across eastern, central, and western regions reported that 72.21% of students were physically inactive (Yu et al., 2022), and a survey of several colleges in Jiangsu Province found that over 80% of students exercised less than 1 h per day, with only 29.9% engaging in extracurricular physical activity three or more times per week (Ding et al., 2019). These findings contextualize the low PA levels observed in our sample, underscoring the urgency of promoting both PA and PAE to improve adolescent physical fitness.

Moreover, utilizing a longitudinal study design, Kruk et al. (2018) offered new insights into the relationship between PAE and moderate-to-vigorous physical activity (MVPA). Their findings demonstrated that PAE precedes MVPA, lending support to the hypothesis that “enjoyment increases MVPA.” The findings that support the hypothesis “enjoyment increases moderate-to-vigorous physical activity (MVPA)” are in line with the broaden-and-build theory of positive emotions (Fredrickson, 1998, 2013). By synthesizing the findings from previous studies with the results of the current research, it is clear that enhancing PAE among children and adolescents can effectively stimulate their intrinsic motivation. Encouraging them to take the initiative in improving their own PA levels is likely to lead to significant enhancements in PF with long-term and lifelong benefits.

This study has several limitations. First, the PA measurements were based on a subjective questionnaire, which may introduce recall bias. Future research should combine objective measures like accelerometers or other wearable devices with validated self-reports to balance feasibility and precision. Second, the participants were all from the same province in China, and the economic and cultural differences across various geographic regions could affect the results. Therefore, future research should aim to broaden the survey’s scope to address the potential impact of geographic variability.

## Conclusion

PAE is positively associated with PF, with PA partially mediated the relation between PAE and PF. This suggests that neglecting the development of PAE in adolescents may undermine efforts to improve PF through increases in PA levels. To effectively encourage adolescents to participate in PA, it is crucial to prioritize enriching their exercise experiences and nurturing an intrinsic enjoyment of physical activity. By enhancing PAE, adolescents are more likely to achieve meaningful and high-quality increases in PA. These improvements not only have immediate benefits but also foster long-term positive effects, ultimately leading to significant enhancements in their PF.

## Data Availability

The raw data supporting the conclusions of this article will be made available by the authors, without undue reservation.
