# Correction: Why have extensive efforts to improve adolescents' physical fitness seen limited success? A mediation analysis of physical activity enjoyment, physical activity and physical fitness

**DOI:** 10.3389/fpsyg.2025.1700234

**Published:** 2025-09-19

**Authors:** Jiale Peng, Wenlang Yu, Wenxing Wang, Leyi Wang, Wei Shan, Hong Ren

**Affiliations:** ^1^College of Physical Education, Guangdong University of Education, Guangzhou, China; ^2^School of Sport Science, Beijing Sport University, Beijing, China; ^3^Key Laboratory of Exercise and Physical Fitness (Beijing Sport University), Ministry of Education, Beijing, China; ^4^Sports Coaching College, Beijing Sports University, Beijing, China; ^5^China Institute of Sport and Health Science, Beijing Sport University, Beijing, China

**Keywords:** exercise, exercise enjoyment, teenager, mediation analysis, health promotion

Affiliation College of Physical Education, Guangdong University of Education, Guangzhou, China was omitted for author Jiale Peng. This affiliation has now been added for author Jiale Peng.

The original version of this article has been updated.

